# Case report: Anti-CARPVIII autoantibody-associated mixed dementia

**DOI:** 10.3389/fpsyt.2023.1133302

**Published:** 2023-05-05

**Authors:** Niels Hansen, Bianca Teegen, Sina Hirschel, Jens Wiltfang, Björn H. Schott, Berend Malchow, Bartels Claudia

**Affiliations:** ^1^Department of Psychiatry and Psychotherapy, University Medical Center Göttingen, Goettingen, Germany; ^2^Translational Psychoneuroscience, Department of Psychiatry and Psychotherapy, University Medical Center Goettingen, Goettingen, Germany; ^3^Clinical Immunological Laboratory Prof. Stöcker, Groß Grönau, Germany; ^4^German Center for Neurodegenerative Diseases (DZNE), Goettingen, Germany; ^5^Neurosciences and Signaling Group, Institute of Biomedicine (iBiMED), Department of Medical Sciences, University of Aveiro, Aveiro, Portugal; ^6^Leibniz-Institute of Neurobiology, University of Magdeburg, Magdeburg, Germany

**Keywords:** anti-CARPVIII autoantibody, dementia, psychiatry, autoimmunity, mixed dementia

## Abstract

**Background:**

Anti-carbonic anhydrase-related protein VIII (CARPVIII) is reported to be associated with paraneoplastic cerebellar degeneration. Our case extends the spectrum of anti-CARPVIII-associated disease to severe cognitive impairment.

**Methods:**

We present the case of a 75-year-old woman who presented to our Department of Psychiatry and Psychotherapy with a dementia syndrome. The diagnostic approach included magnetic resonance imaging (MRI), cerebrospinal fluid analysis (CSF) analysis involving autoantibody determination, and neuropsychological examination.

**Results:**

Neuropsychological examination revealed severe cognitive impairment meeting the criteria for dementia. MRI showed evidence of moderate cerebral microangiopathy. CSF analysis revealed mild pleocytosis, and serum analysis revealed anti-CARPVIII autoantibodies. Based on the dementia syndrome entailing signs of CNS inflammation such as pleocytosis and the repeated detection of anti-CARPVIII autoantibodies in serum, we diagnosed autoimmune dementia as a component of mixed dementia with additional vascular dementia components.

**Conclusion:**

Our finding adds severe cognitive impairment to the spectrum of anti-CARPVIII-associated disease. However, detecting anti-CARPVIII antibodies may also be an incidental finding in conjunction with typical mixed dementia. Further studies are needed to evaluate the relevance of these clinical findings.

## 1. Introduction

Anti-carbonic anhydrase-related proteins such as anti-carbonic anhydrase-related protein VIII (CARPVIII) were detected in paraneoplastic cerebellar degeneration (1–3). Carbonic anhydrase-related proteins play a role in various biological processes (4). CARPV III is a catalytically active protein in the carbonic anhydrase (CA) gene family; it has been associated with colorectal cancer (5) and breast cancer (6). There has been no report to date of progressive cognitive impairment associated with CARPVIII autoantibodies. We present the case of a 75-year-old woman with a dementia syndrome associated with CARPVIII autoantibodies. In addition to her dementia’s possible autoimmune origin we detected circumstantial evidence of a vascular component.

## 2. Case presentation

**Figure 1:**
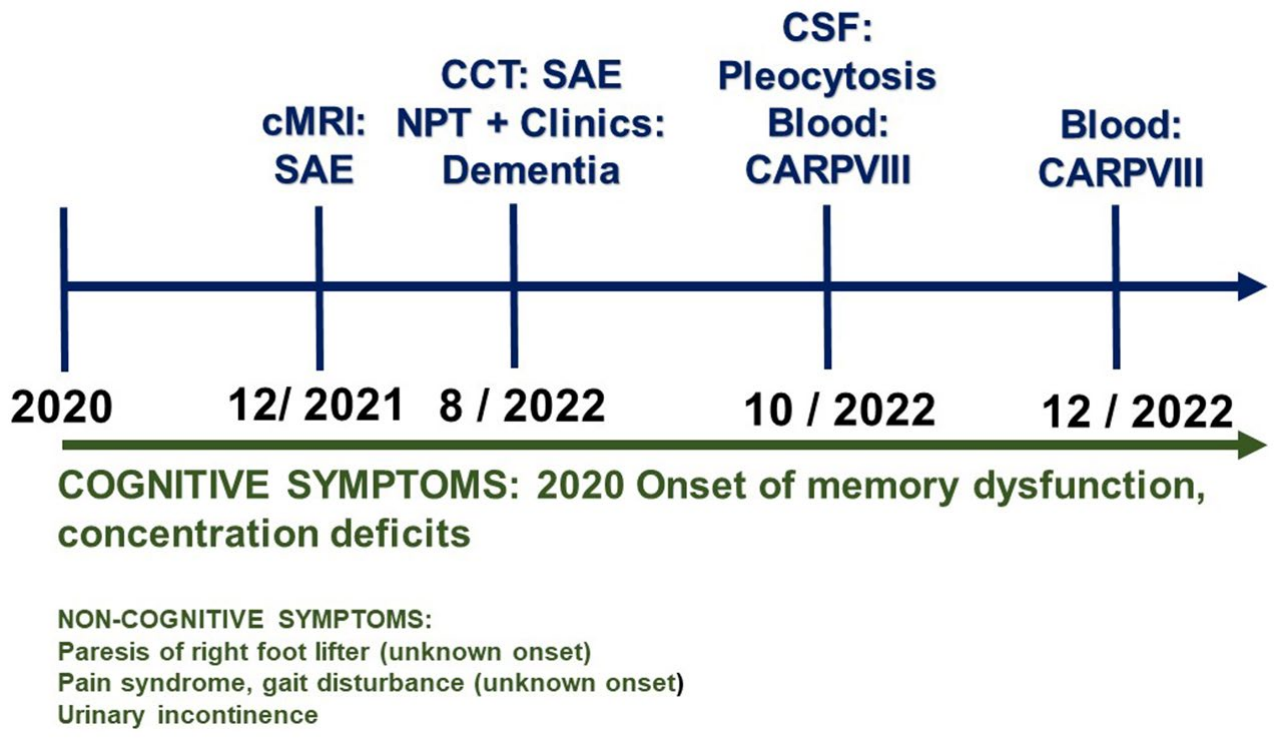
Time course of the patient’s symptoms and diagnostics. CARPVIII, Anti-carbonic anhydrase-related protein VIII; MRI, magnetic resonance imaging; NPT, neuropsychological testing; SAE, subcortical arteriosclerotic encephalopathy.

**Figure 2:**
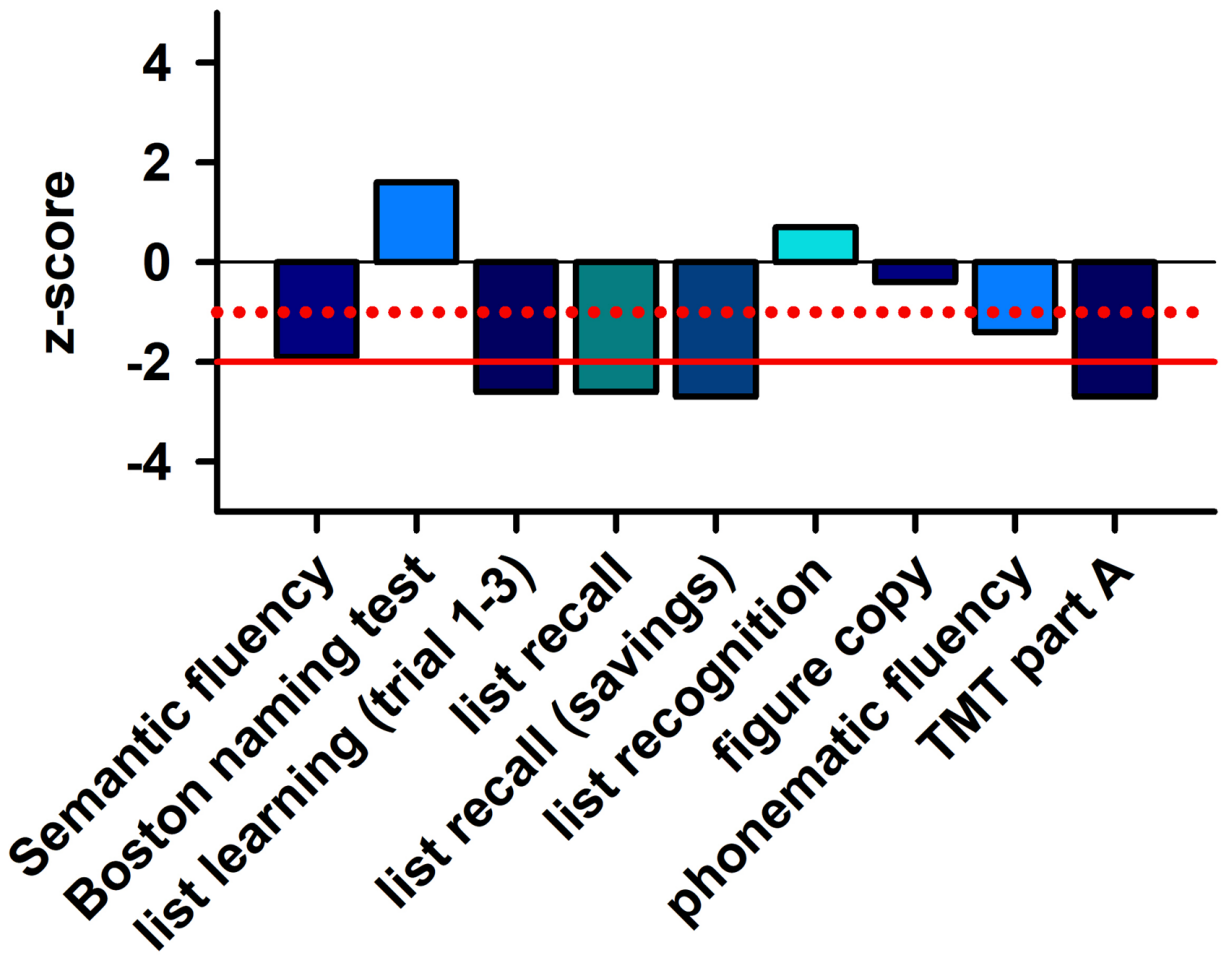
Neuropsychological profile of patient. TMT part A, trial making test part A. The dotted red line indicates the level of the single standard deviation and the solid red line shows the level of the double standard deviation.

A 75-year-old woman presented to the memory outpatient clinic of the Department of Psychiatry and Psychotherapy of the University Medical Center Göttingen because of concentration difficulties as well as memory and retentiveness disorders that had been gradually developing for 2 years (Table 1; Figure 1). She could not always remember orders for certain activities. No episodes of transient loss of consciousness have been reported. Similarly, there was no clinical evidence of delirium. She also complained of a gait disorder, but is unsure about when exactly it started. Her recurrent depressive disorder had been documented; she currently suffers mild to no specific depressive symptoms involving reduced drive, impaired concentration, brooding cognitions, apathy, and social withdrawal. The patient is already dependent on her grandson’s help, as he lives with her in the house. She was a saleswoman, and worries a lot about her daughter. Her psychopathological examination confirmed the reported disturbances of memory and retention, but there was no other psychopathology evident. Neurological examination revealed an extinguished patellar reflex on the right side and extinguished Achilles tendon reflexes on both sides. She also revealed mild foot jack paresis on the right. We found no evidence of rigor, tremor, or cogwheel phenomenon. She also complained of urinary incontinence and occasional fecal incontinence. Pre-existing conditions are a chronic pain syndrome with psychological and somatic factors of unclear onset. She has ventrolisthesis of LWK3 versus LWK4 and LWK4 versus LWK5, in addition to facet joint atresia and osteochondrosis intervertebralis. In addition, she has two artificial knee joints, as well as arterial hypertension, obesity, and presbycusis. She suffered a Weber B fracture on the left side in 2017. Neuropsychological testing revealed impaired semantic and phonematic word fluency, reduced cognitive processing speed, faulty switching ability, a pathological clock test, and verbal memory deficits (encoding and delayed recall of verbal, non-associated information, encoding and delayed recall of complex verbal content) (Figure 2). Results from her thorough neuropsychological tests are in Figure 2. The other parameters tested, such as confrontation recognition, action planning, working and figural memory, verbal discriminability, visuoconstructive skills, and visuospatial perception, corresponded to her educational level. Her profile was thuson the borderline between mild amnestic cognitive impairment and an incipient dementia syndrome. Taking into account her significantly impaired daily living skills (B-ADL = 7.8) (according to her daughter), the dementia criteria are clinically fulfilled. Her magnetic resonance imaging (MRI) findings (Table 1) revealed a Fazekas grade II and medial temporal lobe atrophy score of 2 suggesting mixed dementia. In more detail: the MRI examination she had December of last year showed evidence of a general, primarily internal cerebral volume reduction with an advanced microangiopathic damage pattern. We detected Fazekas grade II microangiopathic anomalies. When she underwent MRI, her cognitive symptoms had been present for 1 year. The computed tomography taken 1 year later (2 months after she presented in our outpatient memory clinic) also showed a dilated ventricular system with widespread brain atrophy. Her (123) I N-omega-fluoropropyl-2beta-carbomethoxy-3beta-{4-iodophenyl}nortropane single photon emission computed tomography examination showed no reduced dopaminergic uptake in the nigrostriatal system. A differential-diagnostic cerebrospinal fluid (CSF) puncture showed mild pleocytosis (6/μL, reference: ≤ 5/μL, Table 1) with a predominant pattern of lymphocytes (93%), elevated phosphorylated tau protein 181 (54.8 pg/mL, pathological: > 50 pg/mL, Table 1) and total tau protein (418 pg/mL, reference, <415 pg/mL), and anti-CARPVIII autoantibodies in the serum at 1:32 intensity (Table 1; Figure 1). The CSF analysis revealed no abnormalities in the beta amyloid peptides: amyloid beta 42 (Aß42), amyloid beta 40 (Aß40) and the amyloid beta 42/40 (Aß42/40) ratio were all in the normal range. In a repeated blood test 2 months later, we detected anti-CARPVIII autoantibodies again at a greater intensity of 1:32 (Figure 1). The autoantibodies were determined in the Clinical Immunological Laboratory from Prof. Stöcker. Relying on the dementia syndrome involving signs of CNS inflammation such as pleocytosis and after detecting anti-CARPVIII autoantibodies in serum, we diagnosed an autoimmune dementia as a component of mixed dementia with additional vascular dementia components present. Our repeated detection of anti-CARPVIII autoantibodies in her serum confirmed their presence as a relevant finding. Portions of the dementia syndrome may also be vascular in origin. There was no evidence of dementia with Lewy bodies or dementia due to Parkinson’s disease. In case of an inconspicuous ratio Aß42/40, an Alzheimer pathology as a second etiology of mixed dementia is equally unlikely, but cannot be excluded in the presence of elevated ptau181. Thus, we cannot entirely rule out a typical mixed dementia with both vascular components and Alzheimer pathology, but that is quite unlikely. Furthermore, as her ptau181 was only slightly elevated, Alzheimer’s disease is unlikely as the cause of her cognitive impairment. She suffered from a gait disturbance, urinary incontinence, and dementia-like syndrome, thus fulfilling the Hakim triad, so we considered the differential diagnosis of normal pressure hydrocephalus. However, there was no evidence of normal pressure hydrocephalus on imaging, and the gait disturbance could be attributable to her pre-existing spinal anomalies and foot lifter paresis. Her cognitive impairments could also be explained differently *via* the mixed dementia, thus ruling out a normal pressure hydrocephalus as a differential diagnosis. Our patient underwent her last follow-up examinations as an inpatient in December last year. She has not undergone any additional follow-up examinations since then. Therapeutically speaking, we limited ourselves to treating her vascular risk factors by prescribing telmisartan 80 mg/d and atorvastatin 20 mg/d. She has not been offered immunotherapy given the slow progression of her symptoms and mixed dementia. However, should her symptoms begin again to deteriorate and were we to detect anti-CARPVIII autoantibodies again, immunotherapy would be an option as an individualized curative approach.

**Table 1:** Clinical and laboratory characteristics of patient.

Parameter	
Demographic parameter
Sex	Female
Age y	75
Age of onset y	73
Psychopathology
Orientation dysfunction	0
Attentional dysfunction	1
Memory disturbances	1
Formal thought disorder	0
Affective disturbance	0
Drive and psychomotor disturbance	0
CSF
Cell count (<5 μL)	6
Albumin mg/L	273
Tau protein (<415 pg/mL)	418
P Tau protein 181 (<50 pg/mL)	54.8
Aß42 (>570 pg/mL)	1,218
Aß40	13,283
Ratio Aß42/40 (>0.06)	0.092
Blood brain barrier disturbance	0
Intrathecal IgG synthesis	0
Serum autoantibodies	Anti-CARPVIII++1: 32
MRI
Generalized atrophy	1
Focal atrophy	1
Hippocampal atrophy	1
Vascular pathology	1

Aß42, Amyloid-ß 42; Aß40, amyloid-ß 40; CSF, cerebrospinal fluid; MRI, magnetic resonance imaging; P Tau Protein 181, phosphorylated tau protein 181; ratio Aß42/40, ratio of amyloid-ß 42/ ratio of amyloid-ß 40; y, years. The values are depicted as mean ± standard deviation. For laboratory data normal ranges are shown in brackets. Reference values: refers to reference values from the Laboratory for Clinical Neurochemistry and Neurochemical Dementia Diagnostics, Department of Psychiatry and Psychotherapy, University Hospital Erlangen. *0 = item not present, # 1 = item present.

## 3. Discussion

Our results demonstrate the novelty of a progressive cognitive impairment associated with CARPVIII autoantibodies. CARPs are involved in acid–base regulation and motor coordination, as studies in zebrafish showed (7). In particular, CARPVIII is involved in motor coordination functions in mice and humans (8). Interestingly, a homozygous missense mutation in CARPVIII protein impaired the function of inositol 1,4,5-trisphosphate to bind to inositol 1,4,5-trisphosphate receptor I, resulting in ataxia associated with mild cognitive impairment (9). Inositol 1,4,5-trisphosphate, which binds to the inositol receptor, is important in regulating the intracellular calcium concentration as a calcium channel in the endoplasmic reticulum membrane. In addition, inositol 1,4,5-trisphosphate, which binds to the inositol receptor, is very abundant in Purkinje cell neurons. Because the cerebellum is also involved in cognitive processes through anatomical fiber projections to the cortex and hippocampus (10), it is conceivable that cognition may be chronically impaired in patients with persistent CARPVIII autoantibodies due to dysfunctional connectivity between the cerebellum and hippocampus. Another possibility is that preexisting vascular dementia characterized by severe cerebral microangiopathy is exacerbated by an inflammatory process detected by CARPVIII antibodies and pleocytosis. Autoantibodies have even been detected in classic forms of dementia such as Alzheimer’s disease (11) and Creutzfeld-Jacob disease (12). Vascular dementia is reportedly associated with antiphospholipid antibodies (13). Thus, in our patient, a mixture of autoimmune and vascular processes is probably responsible for her worsening cognitive functions, although we cannot demonstrate a direct causal relationship. Viral CARPs, for example, have the important function of binding to host cells. Therefore, autoantibody production could also be directed against the process of virus docking to the host cell. In this sense, anti-CARPVIII autoantibodies may reflect a mechanism in our immune system’s antiviral defense strategy.

Weaknesses in our case report include the low evidence level inherent to a case report. In addition, as we detected no CARPVIII antibodies in the CSF and also found no evidence of intrathecal IgG synthesis, our claim that she may have an autoimmune-related cognitive disorder is somewhat weakened. Ultimately, it is unclear whether the vascular anomalies are contributing substantially to her cognitive disorder. Strengths of our report are that, to our knowledge, anti-CARPVIII autoantibodies have not previously been reported in association with cognitive disorder. Furthermore, the fact that we detected anti-CARPVIII antibodies repeatedly suggests that this is a relevant finding. We therefore believe that this case report’s novelty represents a clear strength – and one that outweighs the weakness of a low evidence level. The anti-CARPVIII autoantibody has extremely seldom been reported in a clinical context, but it has already being investigated in a few patients in research studies addressing neuroimmunological disorders (2, 14).

Our case highlights the need for further research on the occurrence of anti-CARPVIII autoantibodies in dementia and, in particular, in mixed dementia coinciding with vascular pathology. The prognosis is unclear, as we have no experience with anti-CARPVIII associated cognitive impairment. The vascular damage with no evidence of Alzheimer’s pathology suggests a rather slow progression, which, however, could also be stepwise and not quite uniform. Thus, the outcome with the present course is rather favorable with regard to an incipient need for care and a rapid loss of independence. However, the possibility of a typical mixed dementia involving neurodegenerative and vascular pathology cannot be excluded, and our detection of CARPVIII autoantibodies might thus be an incidental finding. In conclusion: our report broadens the spectrum of autoantibodies occurring in autoimmune dementia and mixed dementia with vascular co-pathology.

## Data availability statement

The raw data supporting the conclusions of this article will be made available by the corresponding author, without undue reservation.

## Ethics statement

The study involving human participants was reviewed and approved by Ethics committee of the University Medical Center Göttingen. The patient provided their written informed consent to publish the clinical data. Written informed consent was obtained from the individual for the publication of any potentially identifiable images included in this article.

## Author contributions

NH wrote the article. All authors listed have made a substantial, direct, and intellectual contribution to the work and approved it for publication.

## Conflict of interest

The authors declare that the research was conducted in the absence of any commercial or financial relationships that could be construed as a potential conflict of interest.

## Publisher’s note

All claims expressed in this article are solely those of the authors and do not necessarily represent those of their affiliated organizations, or those of the publisher, the editors and the reviewers. Any product that may be evaluated in this article, or claim that may be made by its manufacturer, is not guaranteed or endorsed by the publisher.
